# Tuberculosis suggesting lung cancer

**DOI:** 10.1590/0037-8682-0403-2025

**Published:** 2026-02-06

**Authors:** Bruno Hochhegger, Gláucia Zanetti, Edson Marchiori

**Affiliations:** 1 Department of Radiology, University of Florida, Gainesville, Florida, USA.; 2 Departamento de Radiologia, Universidade Federal do Rio de Janeiro, Rio de Janeiro, RJ. Brasil.

**FIGURE 1:**
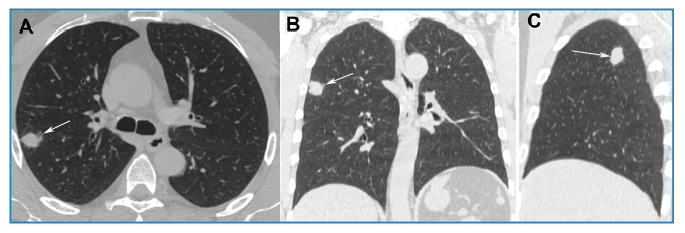
Non-enhanced chest CT images with axial **(A)**, coronal **(B)**, and sagittal **(C)** reconstruction reveal a nodule with homogenous soft-tissue density, irregular contours, and a maximum diameter of approximately 19 mm in the upper lobe of the right lung (arrows).

**FIGURE 2:**
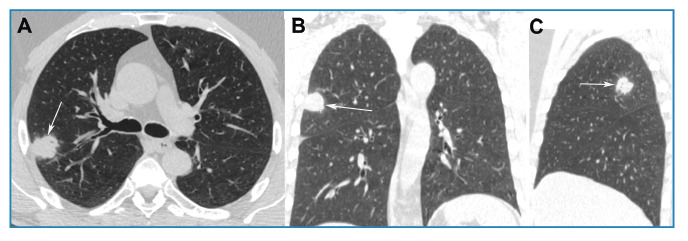
Control chest CT images with axial **(A)**, coronal **(B)**, and sagittal **(C)** reconstruction, obtained 6 months after those in Figure 1, demonstrate nodule growth, with a diameter of approximately 25 mm (arrows).

**FIGURE 3:**
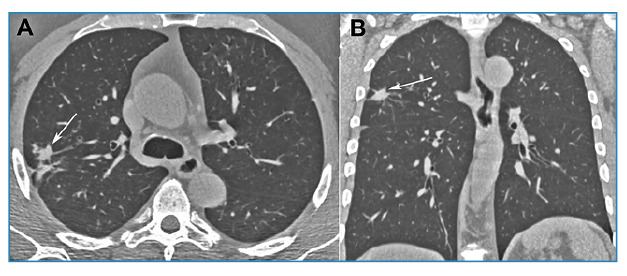
Control chest CT images with axial **(A)** and coronal **(B)** reconstruction, obtained 1 year after the start of treatment, show only residual fibrotic lesions (arrows).

A 56-year-old Brazilian man with a history of smoking and no respiratory symptoms underwent chest radiography on admission, which revealed a pulmonary nodule. Chest computed tomography (CT) further showed a soft tissue density nodule in the right upper lobe (Figure 1). Bronchoalveolar lavage culture was negative for tuberculosis and fungi, and follow-up was recommended for the patient. A follow-up CT scan performed six months later showed that the nodule had grown (Figure 2). Positron emission tomography-computed tomography (PET-CT) demonstrated that the nodule had a high uptake (SUV_max_ = 8.2), and surgery was indicated. Following admission, the patient developed a productive cough and subsequently underwent an acid-fast bacillus test, which returned positive. Accordingly, surgery was suspended and tuberculosis treatment was initiated. A one-year follow-up CT scan revealed residual fibrotic lesions (Figure 3). The final diagnosis was confirmed as tuberculosis.

Solitary pulmonary nodules (SPNs) are frequently detected on chest radiographs and CT scans, posing a diagnostic challenge. The differential diagnosis for an SPN includes both malignant conditions, such as primary lung cancer, and benign conditions, such as infections and benign tumors. Management options for SPNs encompass follow-up CT scans, PET-CT scans, and additional invasive procedures to obtain tissue biopsies for diagnosis. The utility of PET-CT in identifying the cause of lung nodules is highly debated due to its limited specificity in areas endemic for infectious lung diseases. In regions with endemic granulomatous diseases, such as Brazil, the specificity of PET-CT for lung cancer diagnosis is 40%^1^. These findings do not fully support the use of PET-CT for diagnosing lung cancer in endemic regions^1^ ^-^ ^3^.
